# Supplementary material for: Dissecting the circuit for blindsight to reveal the critical role of pulvinar and superior colliculus
Source: Nat Commun. 2019 Jan 11;10:135. doi: 10.1038/s41467-018-08058-0 (PMC6329824; doi:10.1038/s41467-018-08058-0)
Supplement: Supplementary file 1 — Supplementary Information [file 41467_2018_8058_MOESM1_ESM.pdf]

Supplementary Information

Dissecting the circuit for blindsight to reveal the critical role of pulvinar and superior colliculus

Kinoshita et al.

Supplemental Inventory

Supplementary Figure 1 (related to Fig. 1). Extent of V1 lesion.

Supplementary Figure 2 (related to Fig. 3). Individual results of vlPul inactivation by muscimol injections.

Supplementary Figure 3 (related to Fig. 3). Effect of the vlPul inactivation in the visual field affected by V1 lesion and in the lower left visual field, which was considered to be unaffected by the lesion, in Monkey-H.

Supplementary Figure 4 (related to Fig. 4). The intensity profile of MRI image.

Supplementary Figure 5 (related to Fig. 5). Experimental design for the selective synaptic transmission blockade of the pathway from SC to vlPul.

Supplementary Figure 6 (related to Figs. 2 and 5). The location of the pulvinar neurons orthodromically activated by the microstimulation of the SC, and the injection sites of HiRet vector.

Supplementary Figure 7 (related to Fig. 6). The effect of target contrast on visually guided saccade (VGS) performance before and after Dox administration.

Supplementary Figure 8 (related to Method). The VGS task performances before the 1st and 2nd Dox administration in Monkey-C.

Supplementary Table 1 (related to Fig. 7). Number of CaMKII positive cells in the dLGN of 3 V1 lesioned monkeys.

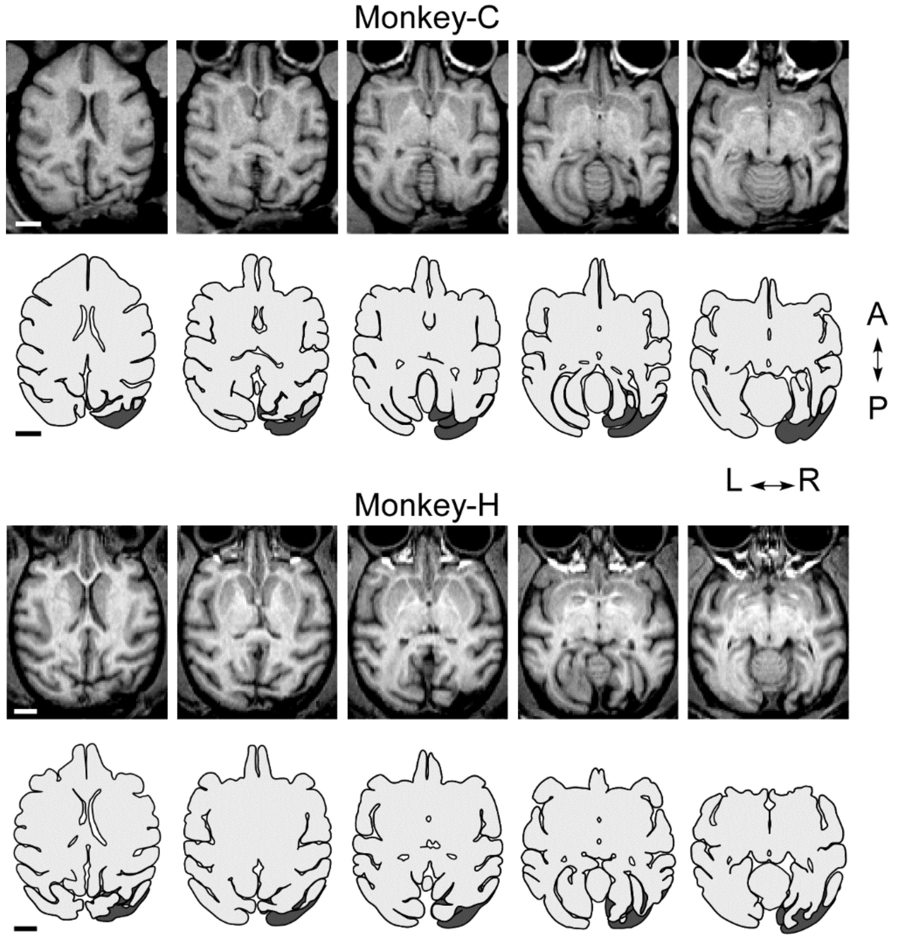

Supplementary Figure 1 (related to Fig. 1). Extent of V1 lesion.

The drawings of brain depicted in Fig. 1A are redisplayed and the MRI images, on which the drawings were based, are shown above. The estimated lesion sites are indicated as dark gray area in the drawings. Scale bar; 1 cm.

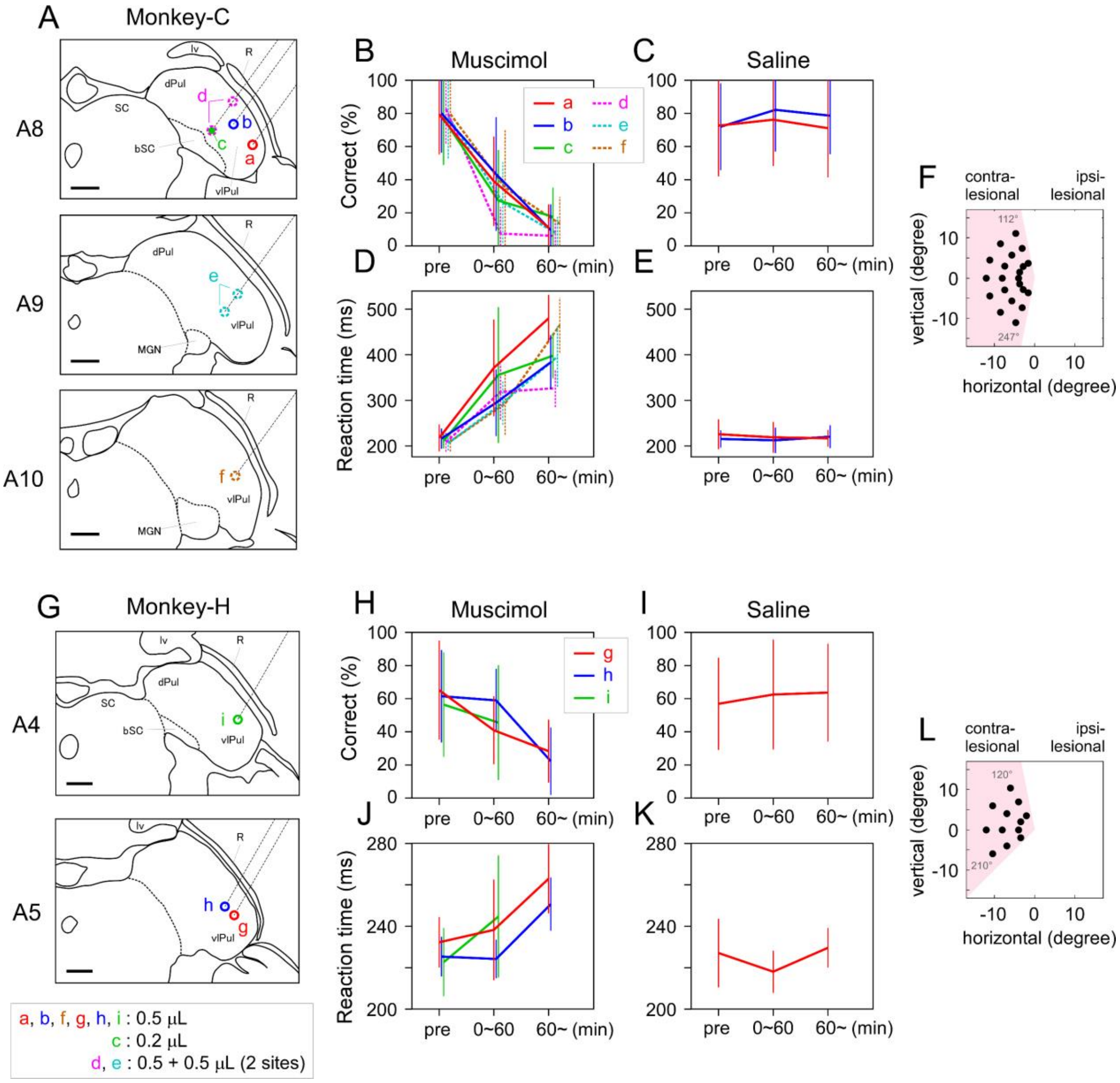

Supplementary Figure 2 (related to Fig. 3). Individual results of vlPul inactivation by muscimol injections

Averaged data of individual experimental days depicted in Fig. 3 are shown for Monkey-C (A-F) and for Monkey-H (G-L). The target contrast was 0.85 for Monkey-C and 0.6 for Monkey-H, respectively. (A and G) The locations of muscimol and saline injections are indicated on the outline drawings of coronal MRI slices as circles marked with small letters (a-i). The injection volume at each site is indicated in the inset below G. At (d) and (e), a couple of injections were done on the same day. At the other sites, a single injection was done on each experimental day. The broken line indicates the penetration of injection needles. The number at left of the drawing indicates position of the slice (e.g. A8 means 8 mm anterior from the ear bar). Scale bar; 2 mm.

(B and H) Deterioration of the visually guided saccade (VGS) performance after the muscimol

injection: The average correct saccade ratios recorded on individual experimental days are plotted for each time window (see red lines in Figs. 3D and 3E) before and after the muscimol injection (0.5 μg per μL). The results of 21 targets (shown in F, Monkey-C) or 12 targets (shown in L, Monkey-H) were averaged for individual days. For each target, the VGS task was repeated 8-10 times (pre), 4-9 times (0-60 min) and 8-11 times (60 min after) for Monkey-C and 16-23 times (pre), 7-23 times (0-60 min) and 11-19 times (60 min after) for Monkey-H. The small letters in the inset of B or H correspond to those in A or G.

(C and I) Effect of saline injections: The correct ratios before and after the saline injections are averaged and plotted in the same manner as in B or H. The color of each plot corresponds to the color in B or H. That is, for example, the results of 0.5 μL muscimol injection at (a) is plotted as red line in B and the results of same volume saline injection at the same site is plotted as same color (red) line in C.

(D and J) Prolonged latencies of the saccade onset after the muscimol injection: The averaged latencies of the saccade onset, recorded on individual experimental days, are plotted. The color of each plot corresponds to the color in B or H.

(E and K) Effect of saline injections: The latencies before and after the saline injection are averaged and plotted in the same manner as in D or J.

(F and L) Black dot indicates the saccade target position used in the analysis for this figure and Fig. 3.

(L) Note that, targets in the lower left direction (240°), in case of Monkey-H, were excluded for the analysis in this figure and shown in Fig. 3, because of the uncertainty in the extent of V1 lesioning corresponding to this area (see Figs. 1C and 1E).

In each graph, error bar indicates standard deviation (SD).

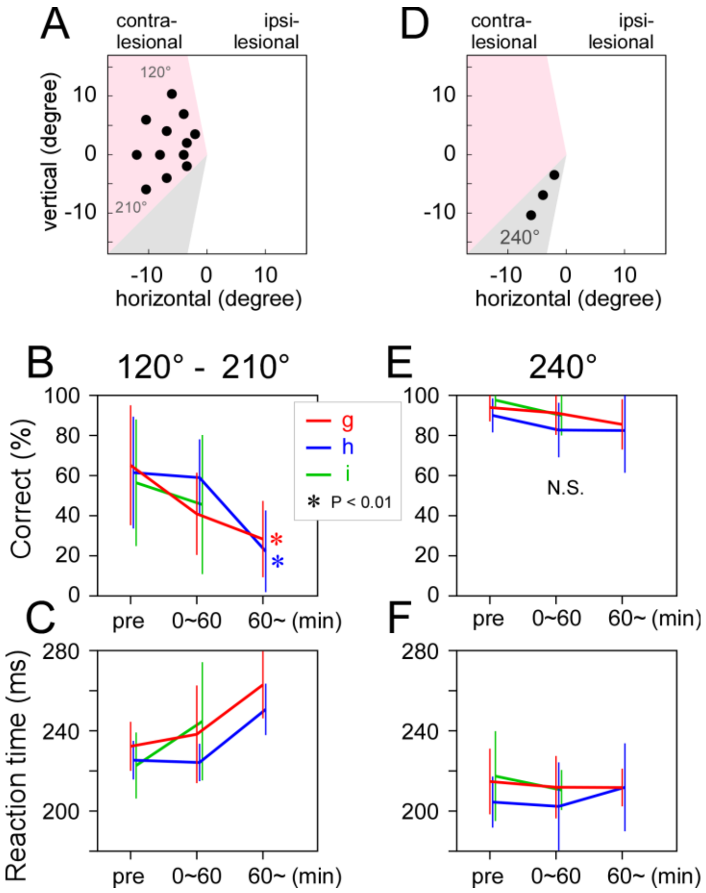

Supplementary Figure 3 (related to Fig. 3). Effect of the vlPul inactivation in the visual field affected by V1 lesion and in the lower left visual field, which was considered to be unaffected by the lesion, in Monkey-H

(A, B and C) A, B and C are the same as Supplementary Figures 2L, 1H and 2J, respectively, to allow for easier comparison with D, E and F.

(D) Black dot indicates the saccade target positions used in the analysis in E and F. Note that, the VGS performance to the dimmed targets presented in this area was not low even after the V1 lesion (Figs. 1C and 1E).

(E and F) The VGS performances (correct ratio (E) and onset latency (F)) are plotted as in the same manner as in B or C. The data recorded in the same day are drawn with same color. Note that, the performance of 60 min after the muscimol injection in the affected field (B) showed significant deterioration ($P < 0.01$, t-test). However, the performance to the lower left targets (E) showed no significant change (N.S.; $P > 0.05$, t-test). For each target, the VGS task was repeated 16-23 times (pre), 7-23 times (0-60 min) and 11-19 times (60 min after) for Monkey-H.

In each graph, error bar indicates SD.

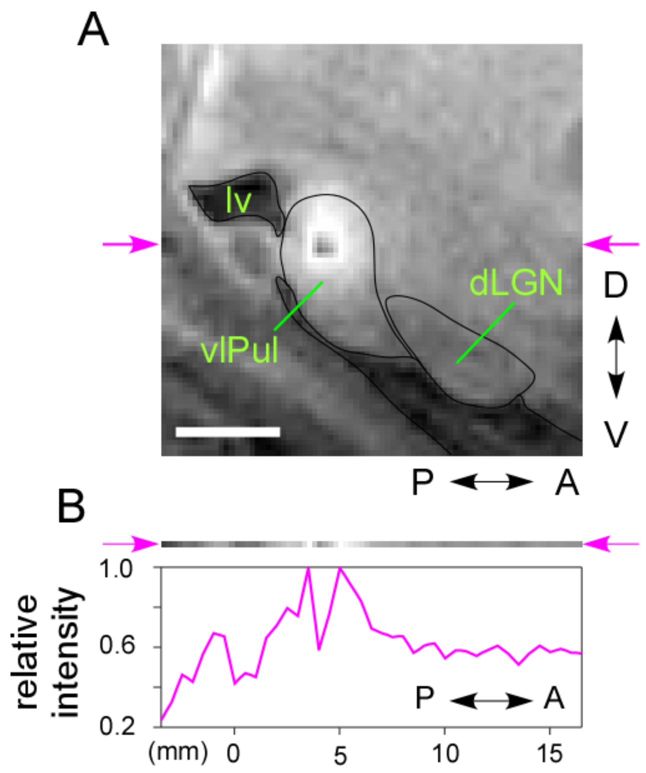

Supplementary Figure 4 (related to Fig. 4). The intensity profile of MRI image

(A) The same parasagittal MRI slice in Fig. 4A is shown here again for the explanation of B. Scale bar; 5 mm.

(B) Relative intensity of image pixels on the line connecting pink arrows in A: X axis indicates the anteroposterior distance from ear bar in mm. The width of strong signal area was about 3 mm. In A, it seems as if there is a black-hole at the center of the strong signal area. The signal strength there, however, was similar to the gray area (e.g., right half of this intensity profile).

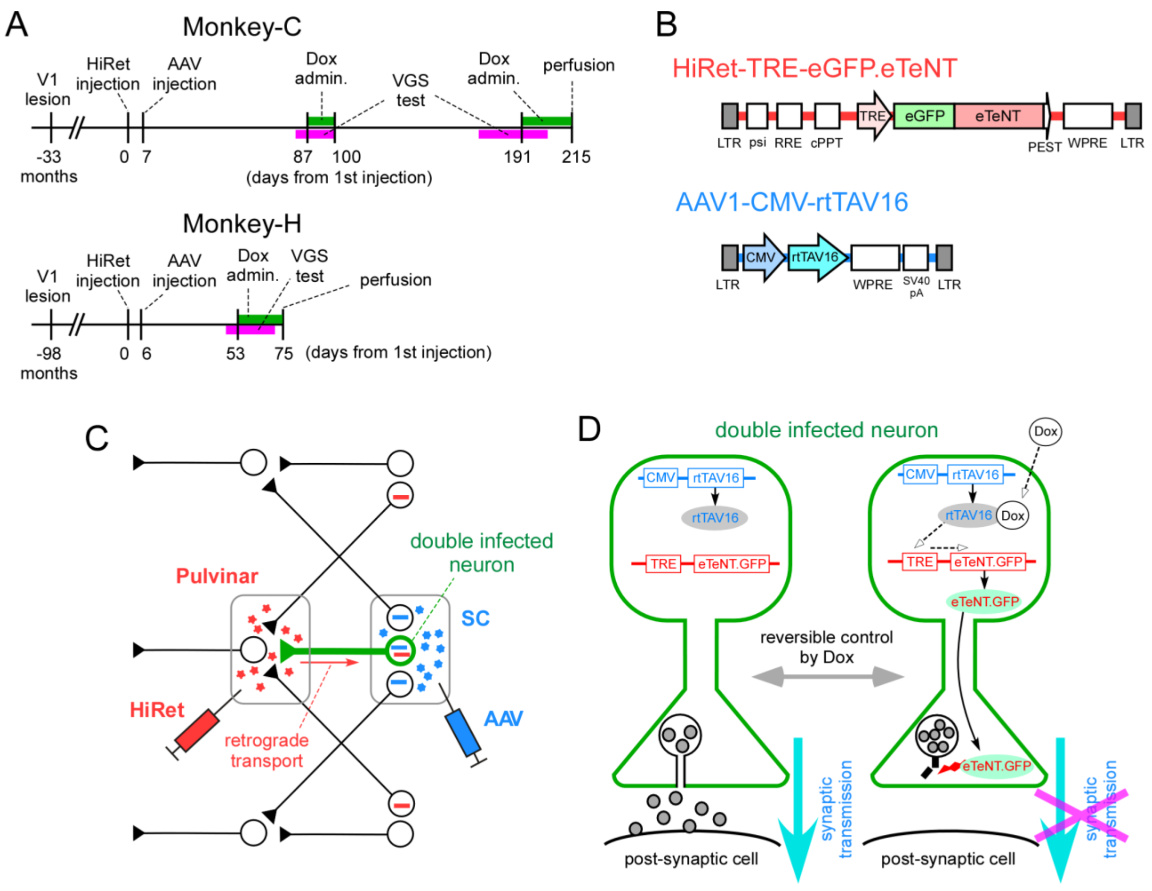

Supplementary Figure 5 (related to Fig. 5). Experimental design for the selective synaptic transmission blockade of the pathway from SC to vlPul

(A) Time course of the pathway selective blockade experiment. Monkey-H was perfused at the end of the first Dox administration period. In Monkey-C, after the termination of first Dox administration and before the second administration period, the performance to the VGS task was tested again.

(B) The construct of viral vectors. HiRet is the highly efficient retrograde gene transfer lentiviral vector. LTR, long terminal repeat; psi, retroviral packaging signal; RRE, Rev responsive element; cPPT, central polypurine tract; TRE, tetracycline responsive element; eGFP, enhanced green fluorescent protein; eTeNT, enhanced tetanus neurotoxin light chain; PEST, PEST sequence, WPRE, Woodchuck hepatitis virus post-transcriptional regulatory element. AAV1 is the adeno-associated virus serotype 1 vector. CMV, cytomegalovirus promoter, rtTAV16, a variant of reverse tetracycline transactivator Tet-on sequence, SV40 pA, Simian vacuolating virus 40 polyadenylation signal.

(C) Schematic drawing of the selective double infection on SC to pulvinar pathway. Each of HiRet or AAV1 could infect the other neurons. However, only the SC neurons projecting to vlPul would be infected by the two vectors.

(D) Schematic drawing of the reversible blockade of synaptic transmission in the double infected

neuron (modified from Kinoshita et al., 2012): In the double infected neurons, Dox and rtTAV16 activate the expression of eTeNT and it cleaves the SNARE protein, which is required for the release of synaptic vesicles. That is, the synaptic transmission of the double infected neuron is suppressed under the administration of Dox.

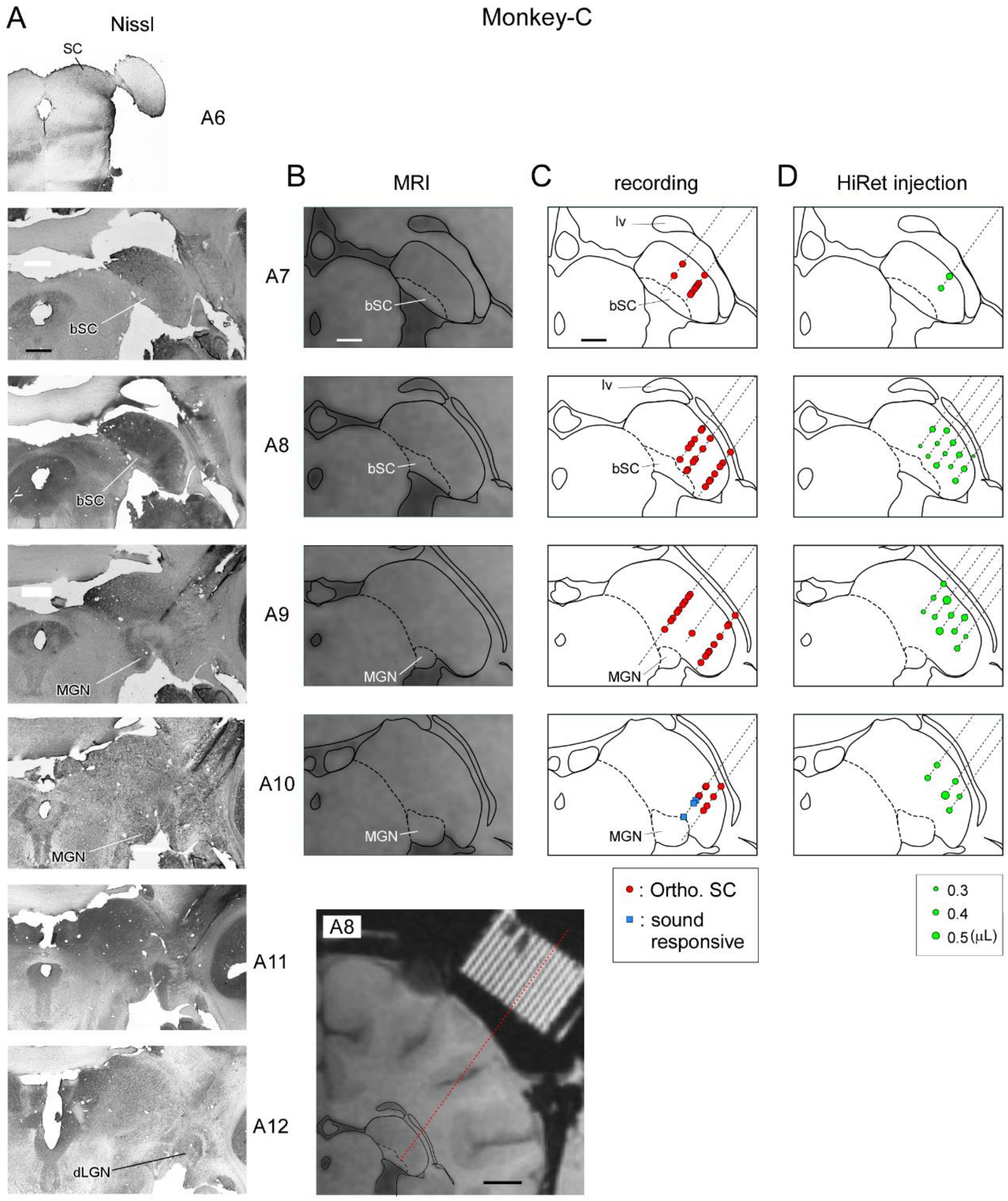

(Supplementary Figure 6 continue)

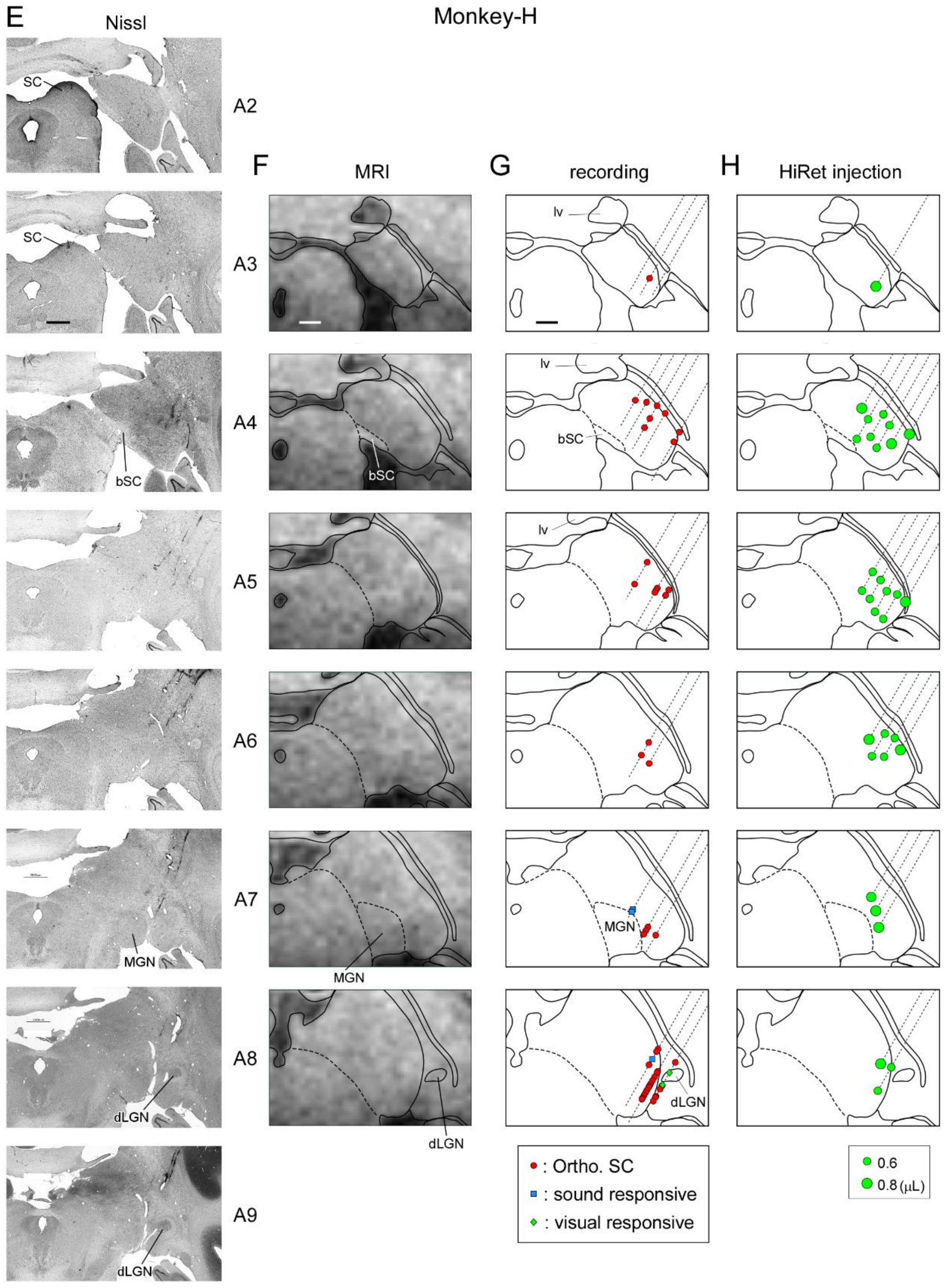

(Supplementary Figure 6 continued)

Supplementary Figure 6 (related to Fig. 2 and Fig. 5). The location of the pulvinar neurons orthodromically activated by the microstimulation of the SC, and the injection sites of HiRet vector

(A and E) The Nissl-stained coronal histological sections. The number at the right indicates anterior position from the ear bar in mm.

(B and F) The coronal MRI images. The line drawings of the brain structures were basically constructed from the MRI images, however, unclear structures were estimated with reference to the adjacent histological sections. The bottom image in (B) shows the representative track (dashed red line) of the electrode or injection needle reconstructed from the MRI image showing the holes of the recording chamber grid used for both recording and injections.

(C and G) The locations of recorded neurons are shown for each monkey ((C) Monkey-C, (G) Monkey-H) on the outlined drawings of MRI images. The red dots indicate recording sites of orthodromically activated neurons to the microstimulation to the superficial layer of SC (presumably mono-synaptic activation, see Fig. 2). The blue rectangle indicates the recording site of unidentified sound responsive activities. The green diamond indicates the recording site of unidentified visual responsive activities. The dashed lines indicate the tracks of recording electrodes.

(D and H) The locations of HiRet injections ((D) Monkey-C, (H) Monkey-H): The size of green dot indicates volume of injection (see inset). The injections were made around the sites of orthodromically activated neurons and were made at least 2 mm apart from the SC. To avoid leakage to the outside of the brain, no injection was made at deep locations.

Scale bar; 2 mm.

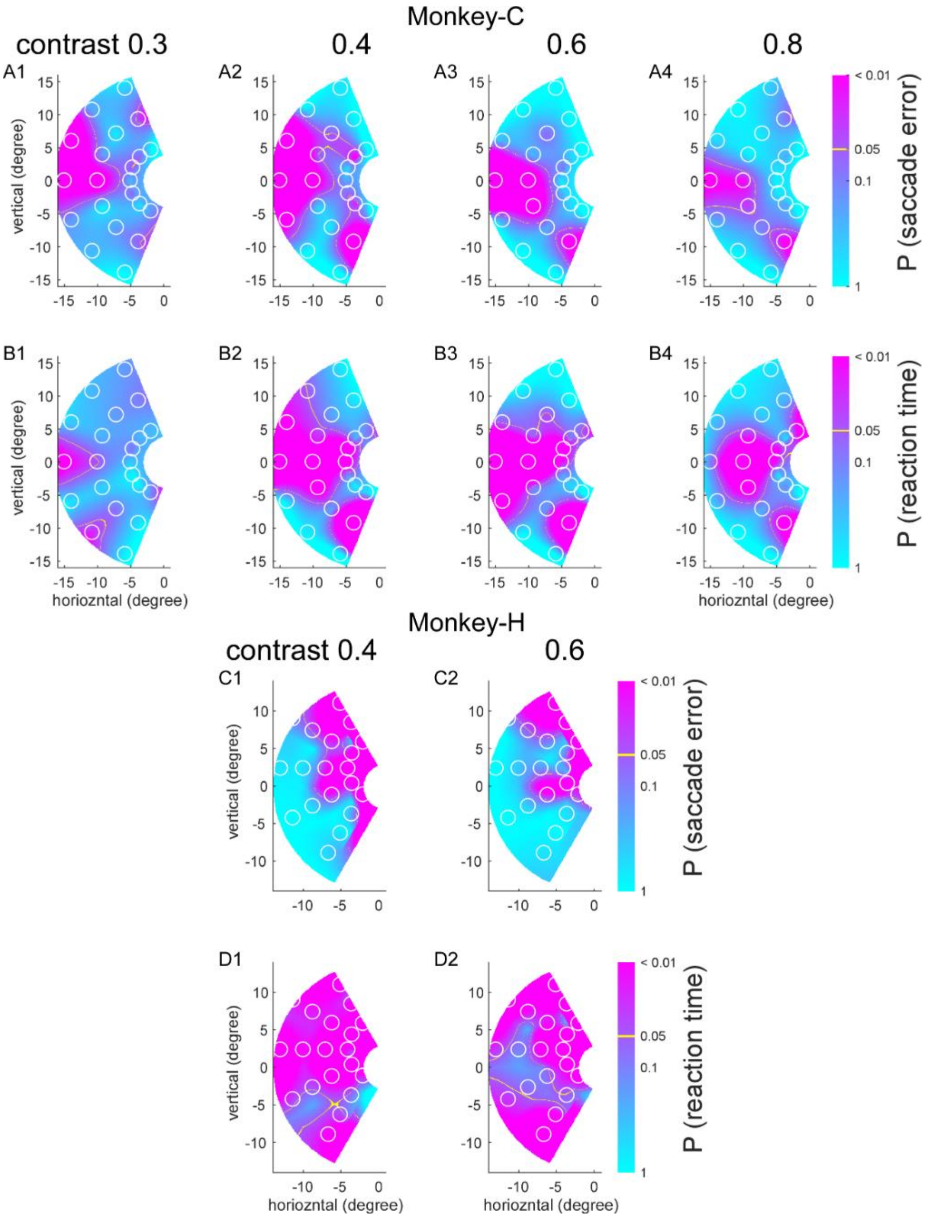

Supplementary Figure 7 (related to Fig. 6). The effect of target contrast on visually guided saccade (VGS) performance before and after Dox administration.

For each target contrast, P value maps of t-test between before administration and during Dox administration, as shown in Fig. 6, were plotted. See Fig. 6, for detail. (A1-4) P value map for the saccade error of Monkey-C. (B1-4) P value map for the reaction time of Monkey-C. (C1-2) for saccade error of Monkey-H. (D1-2) for reaction time of Monkey-H. The target contrast values were 0.3, 0.4, 0.6 and 0.8 for Monkey-C and 0.4 and 0.6 for Monkey-H (indicated on the top). A2, B2, C1 and D1 is same as Fig. 6D, F, J and L, respectively. Number of trials used for t-test at each target location were 42 to 121 for Monkey-C and 206 to 226 for Monkey-H.

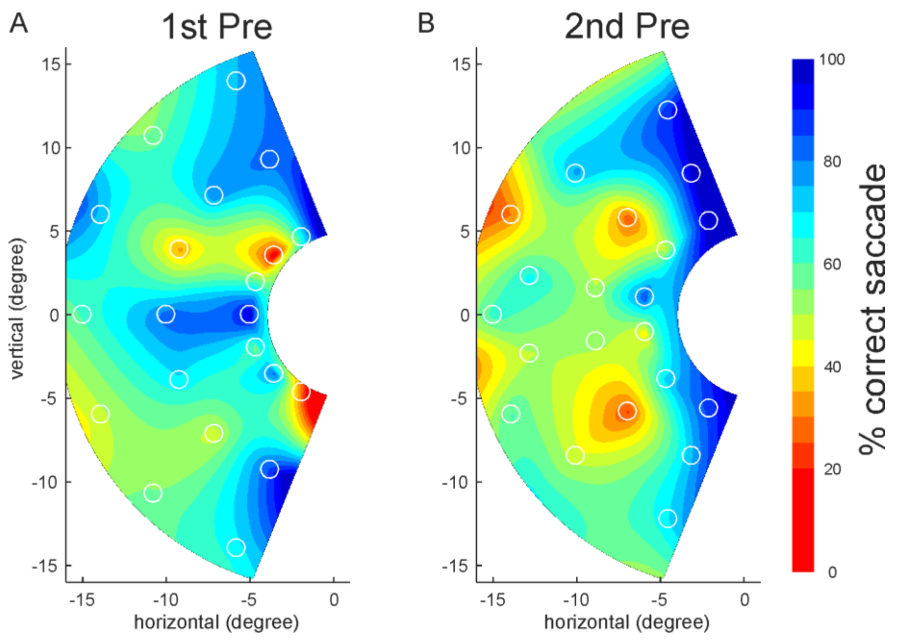

Supplementary Figure 8 (related to Method). The VGS task performances before the 1st and 2nd Dox administration in Monkey-C.

The averaged ratios of correct saccade (that is, a monkey made a saccade into the target window and maintained its gaze. See Method for detail) are mapped on the visual field with the color code shown on the right (note that the blue color indicates higher performance and red indicates lower performance). The values are interpolated between tested locations. (A) For each target location, the averaged correct ratios recorded in four days just before the 1st round of Dox administration (1st Pre; 11 weeks after the vector injections) were mapped for Monkey-C. Target contrast was 0.4. (B) The performance map recorded just before the 2nd round of Dox (2nd Pre; 90 days after the offset of 1st Dox. See Supplementary Figure 5A) for Monkey-C. Target contrast was 0.5. White circle indicates the location of the saccade target. For each target, the number of trials included in this average was 54 – 129. Note that the correct ratios of the 2nd Pre were significantly deteriorated compared with the 1st Pre ($P < 0.05$, one-tailed Student t-test for non-averaged correct ratios, $N = 84$ (1st Pre) and 120 (2nd Pre)), which explains why we discarded the data from the 2nd round (see Methods).

Monkey (survival time)	Ipsilesional dLGN			Contralesional dLGN			Ratio		
	Slide#	K-cells (n)	Area (mm^2)	Slide#	K-cells (n)	Area (mm^2)	K-cells (%)	Area (%)	Density (%)
C (40 months)	161	352	9.1	161	1134	18.2	31	50	62
	171	289	8.2	171	882	17.7	33	46	71
	181	319	7.1	181	814	15.6	39	45	87
H (101 months)	284	144	8.3	284	762	14.4	19	58	33
	304	132	7.0	304	707	15.9	19	44	42
	324	66	4.1	324	442	12.5	15	33	45
A (71 months)	241	319	5.0	295	798	6.5	40	77	52
	263	143	6.6	326	767	12.7	19	52	36
	273	66	4.6	352	824	11.6	8	40	20

Supplementary Table 1 (related to Fig. 7). Number of CaMKII positive cells in the dLGN of 3 V1 lesioned monkeys

In Monkey-C and -H, ipsilesional and contralesional side dLGNs, which are on the same coronal section, are compared. In Monkey-A, parasagittal sections, which have similar laterality, are compared. The months from V1 lesion to perfusion (survival time) is indicated below the initial. Slide#: ID number of the histological section. K-cells: number of the CaMKII positive cells in the section. Area: area of the dLGN (mm^2). Ratio: the ratio of ipsilesional side to the contralesional side (%). Density = the number of K-cells divided by Area. Note that, not only the shrinkage of the dLGN, but also the decrease of number of CaMKII positive cells was found - (The ratio of the cell density was smaller than 100% in all sections). The degree of dLGN degeneration seems to be correlated with the survival time after the lesion.
