# Supplementary material for: Dissecting the circuit for blindsight to reveal the critical role of pulvinar and superior colliculus
Source: Nat Commun. 2019 Jan 11;10:135. doi: 10.1038/s41467-018-08058-0 (PMC6329824; doi:10.1038/s41467-018-08058-0)
Supplement: Supplementary file 3 — Reporting Summary [file 41467_2018_8058_MOESM3_ESM.pdf]

nature research

Corresponding author(s): Tadashi Isa

Reporting Summary

Nature Research wishes to improve the reproducibility of the work that we publish. This form provides structure for consistency and transparency in reporting. For further information on Nature Research policies, see Authors & Referees and the Editorial Policy Checklist.

Statistical parameters

When statistical analyses are reported, confirm that the following items are present in the relevant location (e.g. figure legend, table legend, main text, or Methods section).

n/a	Confirmed	
☐	☒	The exact sample size (n) for each experimental group/condition, given as a discrete number and unit of measurement
☐	☒	An indication of whether measurements were taken from distinct samples or whether the same sample was measured repeatedly
☐	☒	The statistical test(s) used AND whether they are one- or two-sided<br>*Only common tests should be described solely by name; describe more complex techniques in the Methods section.*
☒	☐	A description of all covariates tested
☒	☐	A description of any assumptions or corrections, such as tests of normality and adjustment for multiple comparisons
☐	☒	A full description of the statistics including central tendency (e.g. means) or other basic estimates (e.g. regression coefficient) AND variation (e.g. standard deviation) or associated estimates of uncertainty (e.g. confidence intervals)
☐	☒	For null hypothesis testing, the test statistic (e.g. F, t, r) with confidence intervals, effect sizes, degrees of freedom and P value noted<br>*Give P values as exact values whenever suitable.*
☒	☐	For Bayesian analysis, information on the choice of priors and Markov chain Monte Carlo settings
☒	☐	For hierarchical and complex designs, identification of the appropriate level for tests and full reporting of outcomes
☒	☐	Estimates of effect sizes (e.g. Cohen's d, Pearson's r), indicating how they were calculated
☐	☒	Clearly defined error bars<br>*State explicitly what error bars represent (e.g. SD, SE, CI)*

Our web collection on statistics for biologists may be useful.

Software and code

Policy information about availability of computer code

Data collection	To collect the data in this study, we used the custom codes for Tempo for Windows (v. 10.34, Reflective Computing, USA) and used Spike2 (v.7.10c, Cambridge Electronic Design, Ltd., UK).
Data analysis	To analyze the data and to prepare the figures in this study, we used the custom codes for MATLAB (v. 2016b, MathWorks, Inc., USA) and for Scilab (v. 5.41, Scilab Enterprises, France) and used Spike2 (v.7.10c, Cambridge Electronic Design, Ltd., UK), MRIcron (v. 1, http://people.cas.sc.edu/rorden/mricron/index.html), ImageJ (v. 1.49v, National Institutes of Health, USA) and Canvas (v. 14, ACD Systems of America, Inc., USA).

For manuscripts utilizing custom algorithms or software that are central to the research but not yet described in published literature, software must be made available to editors/reviewers upon request. We strongly encourage code deposition in a community repository (e.g. GitHub). See the Nature Research guidelines for submitting code & software for further information.

Data

Policy information about availability of data

All manuscripts must include a data availability statement. This statement should provide the following information, where applicable:
- Accession codes, unique identifiers, or web links for publicly available datasets
- A list of figures that have associated raw data
- A description of any restrictions on data availability

The data supporting the findings of this study are available from corresponding author upon reasonable request.

Field-specific reporting

Please select the best fit for your research. If you are not sure, read the appropriate sections before making your selection.

☒ Life sciences ☐ Behavioural & social sciences ☐ Ecological, evolutionary & environmental sciences

For a reference copy of the document with all sections, see nature.com/authors/policies/ReportingSummary-flat.pdf

Life sciences study design

All studies must disclose on these points even when the disclosure is negative.

Sample size	No power calculations were performed to determine sample sizes. The sample sizes were determined by the possible number of behavioral task repeat per day and the experimental schedule, which was determined with reference to the former studies (e.g. Kinoshita et al., 2012. Nature)
Data exclusions	No data were excluded from the analysis.
Replication	Replication of the results was confirmed in two of two monkeys. Experiments of two monkeys were conducted in another institutes.
Randomization	In the VGS task, location and contrast of the saccade target was pseudo-randomly chosen from a set of target conditions.
Blinding	The investigators were not blinded in muscimol injection and dox administration experiments. A blinding was not relevant because the task control was conducted not by manually but by the script for TEMPO system.

Reporting for specific materials, systems and methods

Materials & experimental systems

n/a	Involved in the study
☐	☒ Unique biological materials
☐	☒ Antibodies
☒	☐ Eukaryotic cell lines
☒	☐ Palaeontology
☐	☒ Animals and other organisms
☒	☐ Human research participants

Methods

n/a	Involved in the study
☒	☐ ChIP-seq
☒	☐ Flow cytometry
☐	☒ MRI-based neuroimaging

Unique biological materials

Policy information about availability of materials

Obtaining unique materials	The following viral vectors, reported in previous studies (ref. 16, 17, 18, 19), were used: HiRet-TRE-eGFP.eTeNT and AAV1-CMV-rtTAV16. These viral vectors are available from corresponding author upon reasonable request.

Antibodies

Antibodies used	The following commercially available antibodies were used:<br>Primary: Anti-calmodulin-dependent protein kinase IIα antibody (Affinity BioReagents, Golden, CO, USA)<br>Secondary: Goat anti-rabbit IgG (1:200; Vector Laboratories, USA)

April 2018

Validation	The detailed immunostaining procedure, customized from manufacuture's instructions, is described in Method.

Animals and other organisms

Policy information about studies involving animals; ARRIVE guidelines recommended for reporting animal research

Laboratory animals	Two male macaque monkeys (Macaca fuscata and Macaca mulatta, body weight 6.8 and 9.0 kg) were used in this study. Monkey-H is the same animal used in the previous study (ref. 20). The third animal, Monkey-A, which was used only for the immunohistochemistry in this study, is the same animal used in another study (ref. 21).
Wild animals	All macaque monkeys used in this study were supplied from a domestic breeding farm.
Field-collected samples	In this study, no sample was collected from the field.

Magnetic resonance imaging

Experimental design

Design type	Resting state (anesthetized).
Design specifications	*Specify the number of blocks, trials or experimental units per session and/or subject, and specify the length of each trial or block (if trials are blocked) and interval between trials.*
Behavioral performance measures	*State number and/or type of variables recorded (e.g. correct button press, response time) and what statistics were used to establish that the subjects were performing the task as expected (e.g. mean, range, and/or standard deviation across subjects).*

Acquisition

Imaging type(s)	Structural
Field strength	3 tesla
Sequence & imaging parameters	The acquisition sequence was three-dimensional magnetization-prepared rapid-acquisition gradient echo. The voxel size was 0.20-0.82 mm.
Area of acquisition	whole brain

Diffusion MRI ☐ Used ☒ Not used

Preprocessing

Preprocessing software	*Provide detail on software version and revision number and on specific parameters (model/functions, brain extraction, segmentation, smoothing kernel size, etc.).*
Normalization	*If data were normalized/standardized, describe the approach(es): specify linear or non-linear and define image types used for transformation OR indicate that data were not normalized and explain rationale for lack of normalization.*
Normalization template	*Describe the template used for normalization/transformation, specifying subject space or group standardized space (e.g. original Talairach, MNI305, ICBM152) OR indicate that the data were not normalized.*
Noise and artifact removal	*Describe your procedure(s) for artifact and structured noise removal, specifying motion parameters, tissue signals and physiological signals (heart rate, respiration).*
Volume censoring	*Define your software and/or method and criteria for volume censoring, and state the extent of such censoring.*

Statistical modeling & inference

Model type and settings	*Specify type (mass univariate, multivariate, RSA, predictive, etc.) and describe essential details of the model at the first and second levels (e.g. fixed, random or mixed effects; drift or auto-correlation).*
Effect(s) tested	*Define precise effect in terms of the task or stimulus conditions instead of psychological concepts and indicate whether ANOVA or factorial designs were used.*

Specify type of analysis: ☒ Whole brain ☐ ROI-based ☐ Both

Statistic type for inference (See Eklund et al. 2016)	*Specify voxel-wise or cluster-wise and report all relevant parameters for cluster-wise methods.*
Correction	*Describe the type of correction and how it is obtained for multiple comparisons (e.g. FWE, FDR, permutation or Monte Carlo).*

Models & analysis

n/a	Involved in the study
☒	☐ Functional and/or effective connectivity
☒	☐ Graph analysis
☒	☐ Multivariate modeling or predictive analysis
